# Does use of ‘non-trial’ cessation support help explain the lack of effect from offering NRT to quitline callers in a RCT?

**DOI:** 10.1136/tobaccocontrol-2013-051107

**Published:** 2013-07-23

**Authors:** Graeme Docherty, Sarah Lewis, Andy McEwen, Linda Bauld, Tim Coleman

**Affiliations:** 1 Department of Epidemiology and Public Health, UK Centre for Tobacco Control Studies, University of Nottingham, Nottingham, UK; 2 Department of Epidemiology and Public Health, Cancer Research UK Health Behaviour Research Centre, UK Centre for Tobacco Control Studies, University College London, London, UK; 3 UK Centre for Tobacco Control Studies, Institute of Social Marketing, University of Stirling, Stirling, UK; 4 Division of Primary Care, UK Centre for Tobacco Control Studies and NIHR School for Primary Care Research, University of Nottingham, Queen's Medical Centre, Nottingham, UK

**Keywords:** Cessation, Nicotine, Harm Reduction

## Introduction

Quitlines help smokers to stop but few studies have explored how behavioural and medicinal interventions can be optimally delivered via this route.^1^ One of these was the PORTSSS trial, which found that offering free nicotine replacement therapy (NRT) vouchers did not increase cessation rates compared with no offer.^2^ It also found that a ‘proactive’, more intensive call regime from/to clients did not improve cessation rates over ‘usual care’. Was it possible that participants who did not receive a voucher for NRT sought out and used other forms of cessation support, which minimised any effect of receiving the NRT voucher? Use of ‘non-trial’ support varied across PORTSSS trial intervention groups and, in this analysis, we sought to determine whether or not use of this substantially affected trial findings.

## Methods

Our secondary analysis included all 2591 randomised participants of the PORTSSS trial. PORTSSS was a randomised controlled trial (RCT) of an English, government-funded quitline, comparing two forms of behavioural support, with and without the offer of a free NRT voucher using a parallel-group, factorial 2×2 design. Non-trial support used by participants included ‘over the counter’ NRT (n=498; 19.2%), NRT from health professionals (479; 18.5%), bupropion (37; 1.4%), varenicline (165; 6.4%), NHS stop smoking service support (125; 4.8%), NHS one-to-one therapy (221; 8.5%) and a non-NHS quitline (40; 1.5%); any support (978; 37.7%). Binary variables were created for each support type with recipients coded as 1 and non-recipients as 0. We used the same multivariable regression model as in the original trial analysis with the effect of treatment group adjusted for age, gender, age of finishing education and heaviness of smoking, and then additionally adjusted for each of the binary indicators of use of non-trial support to assess whether this altered the effect of treatment.

## Results

A comparison of the two adjusted models (table 1) shows little difference to the trial findings with respect to the primary outcome, prolonged cessation at 6 months (trial model OR 0.86, 95% CI 0.7 to 1.06; additional model OR 0.84, 95% CI 0.66 to 1.07) or any of the secondary outcomes, irrespective of whether self-reported or validated smoking outcomes are used.

**Table 1:** Smoking cessation outcomes in relation to nicotine replacement therapy (NRT)

	Total N=2591	No NRT N=1296	NRT N=1295	Unadjusted OR (95% CI; p value)	Adjusted OR* (95% CI; p value)	Adjusted OR† (95% CI; p value)
Outcomes at 6 months, n (%)						
Prolonged cessation (inc. questionnaire data) (primary outcome)	490 (18.9)	261 (20.1)	229 (17.7)	0.85 (0.70 to 1.04; p=0.11)	0.86 (0.70 to 1.06; p=0.16)	0.84 (0.66 to 1.07; p=0.17)
Carbon monoxide validated prolonged cessation	207 (8.0)	122 (9.4)	85 (6.6)	0.67 (0.50 to 0.90; p=0.008)	0.65 (0.48 to 0.88; p=0.005)	0.63 (0.45 to 0.86; p=0.004)
Self-reported cessation for ≥7 days	531 (20.5)	283 (21.8)	248 (19.1)	0.85 (0.70 to 1.03; p=0.09)	0.85 (0.70 to 1.04; p=0.13)	0.85 (0.67 to 1.07; p=0.17)
Carbon monoxide validated cessation for ≥7 days	200 (7.7)	119 (9.2)	81 (6.2)	0.66 (0.49 to 0.88; p=0.006)	0.64 (0.47 to 0.87; p=0.004)	0.62 (0.45 to 0.86; p=0.004)
Reported cessation for ≥3 months	401 (15.5)	216 (16.6)	185 (14.3)	0.83 (0.67 to 1.03; p=0.09)	0.84 (0.67 to 1.05; p=0.14)	0.86 (0.66 to 1.10; p=0.23)
Reports one or more quit attempts lasting >24 h†	594 (22.9)	289 (22.3)	305 (23.5)	1.07 (0.88 to 1.28; p=0.49)	1.05 (0.86 to 1.27; p=0.60)	1.15 (0.88 to 1.50; p=0.30)
Median (IQR) no. quit attempts reported	2 (1–3)	2 (1–4)	2 (1–3)	n/a	n/a	n/a
Outcomes at 1 month, n (%)						
Prolonged cessation since quit date	1040 (40.1)	520 (40.1)	520 (40.1)	0.99 (0.85 to 1.16; p=0.93)	1.01 (0.86 to 1.19; p=0.88)	1.00 (0.80 to 1.26; p=0.96)
Reported cessation for ≥7 days	831 (32.0)	417 (32.2)	414 (32.0)	0.98 (0.83 to 1.16; p=0.85)	0.99 (0.84 to 1.18; p=0.97)	0.97 (0.77 to 1.22; p=0.80)

*Adjusted for age, gender, educational level and heaviness of smoking index; 2397 cases included in adjusted analyses.

†Additionally adjusted for all forms of non-trial support.

## Discussion

To our knowledge, this is the first analysis to investigate the effect of additional cessation support on the impact of free NRT provision from a quitline. The findings suggest that use of such support does not explain the negative PORTSSS trial findings with respect to NRT. We identified only one other paper investigating associations between quitline outcomes and use of other forms of support^3^; it found that smokers who had used other types of cessation support prior to quitline enrolment were more likely to subsequently stop smoking with quitline help. Little is known about the relative contributions of quitline and non-quitline support to smoking cessation; monitoring and evaluating the relationship of ‘non-trial’ cessation support to outcomes in future quitline studies is important.

**Key messages**

- The PORTSSS trial found that offering free NRT vouchers to quitline clients did not improve cessation rates.
- Use of other cessation support used outside the trial did not explain the negative PORTSSS trial findings with respect to NRT.
